# Probiotic drug interactions in ulcerative colitis: a microbiota and metabolite perspective

**DOI:** 10.3389/fmicb.2026.1944134

**Published:** 2026-09-16

**Authors:** Jana Štofilová, Veronika Benetinová, Izabela Bertková, Zuzana Hanzelová, Anna Kamlárová, Monika Kvaková

**Affiliations:** Center of Clinical and Preclinical Research MEDIPARK, Faculty of Medicine, P.J. Safarik University in Kosice, Kosice, Slovakia

**Keywords:** 5-aminosalicylic acid, biologics, immunomodulators, microbiota, pharmacomicrobiomics, probiotic-drug interactions, ulcerative colitis

## Abstract

Ulcerative colitis (UC) is a chronic relapsing inflammatory bowel disease that imposes a substantial lifelong burden on patients, particularly as it commonly develops during adolescence or early adulthood. Despite major advances in pharmacological management, including the introduction of biologic agents and small-molecule therapies, considerable interindividual variability in therapeutic response remains a major clinical challenge. Emerging evidence in pharmacomicrobiomics indicates that the gut microbiota contributes to this variability through bidirectional interactions with drugs. These interactions involve direct microbial biotransformation of drugs, modulation of host metabolic pathways and transport systems, and pharmacodynamic effects mediated by microbiota that collectively influence drug efficacy, safety, and therapeutic outcomes. Strategies aimed at restoring microbial homeostasis, including dietary interventions, probiotics, prebiotics, synbiotics, postbiotics, and fecal microbiota transplantation, have therefore attracted increasing attention as adjunctive approaches in UC. Among these, probiotics are the most extensively investigated, and selected formulations have demonstrated clinical benefit in inducing and maintaining remission of UC. However, while their direct therapeutic effects have been widely reviewed, considerably less attention has been devoted to understanding how probiotics interact with conventional UC therapies and whether they modify treatment efficacy through pharmacokinetic, pharmacodynamic, or microbiota-mediated mechanisms. This review provides an up-to-date overview of probiotic-drug interactions in UC, with particular emphasis on microbiota- and metabolite-mediated mechanisms that may influence therapeutic efficacy and contribute to interindividual variability in treatment response. Finally, we discuss current concepts for the rational use of probiotics as adjuncts to UC pharmacotherapy and highlight key knowledge gaps and future research directions in this rapidly evolving field. A better understanding of these interactions may ultimately support microbiota-informed therapeutic strategies aimed at optimizing treatment outcomes in UC.

## 1 Introduction

Ulcerative colitis (UC) is one of the two major forms of inflammatory bowel disease (IBD), characterized by recurrent inflammation of the rectal and colonic mucosa that extends continuously from the rectum through variable portions of the colon. The severity of clinical manifestations generally correlates with the extent and intensity of colonic inflammation (Lynch and Hsu, 2025). With an estimated global prevalence of 120.4 cases per 100,000 population and an incidence of 5 cases per 100,000 person-years, UC represents a growing healthcare challenge worldwide (Heydari et al., 2025). Despite its relatively low mortality, UC imposes a substantial burden on patients and healthcare systems, as it typically manifests during adolescence or early adulthood, affecting patients during their most productive years. Recurrent disease flares, gastrointestinal symptoms, fatigue, and the need for lifelong treatment can substantially impair quality of life, social functioning, educational attainment, and professional development (Dahlgren et al., 2022). Consequently, a growing number of individuals require long-term pharmacological treatment, emphasizing the need for strategies that improve therapeutic efficacy while minimizing adverse effects and treatment burden.

A hallmark of UC pathogenesis is disruption of intestinal homeostasis in genetically susceptible individuals, resulting from the interplay between host genetic factors, gut microbiota, epithelial barrier dysfunction, and dysregulated mucosal immune responses (Bu et al., 2025). Aberrant activation of multiple inflammatory signaling pathways, including nuclear factor kappa B (NF-κB), Januse/kinase/signal transducer and activator of transcription (JAK/STAT), mitogen-activated protein kinase (MAPK), and inflammasome-associated cascades, leads to excessive production of pro-inflammatory mediators such as tumor necrosis factor-α (TNF-α), interleukins (IL)-1β, IL-6, IL-13, and interferon-*γ* (IFN-*γ*). This sustained inflammatory response contributes to epithelial barrier dysfunction, chronic mucosal injury, and progressive tissue damage (Jia et al., 2026). Furthermore, long-standing and extensive colonic inflammation is also associated with an increased risk of dysplasia and colorectal cancer (Shinagawa et al., 2026; Kapadia et al., 2025; Rivera et al., 2022). Accumulating evidence suggests that the gut microbiota is not merely a bystander but an active regulator of these signaling networks. Through interactions with epithelial and immune cells and the production of bioactive metabolites, gut microbiota contributes to the maintenance of mucosal immune homeostasis (Zheng et al., 2020). Consequently, alterations in microbial composition and metabolic activity may promote immune dysregulation, amplify inflammatory signaling, and influence both disease progression and therapeutic responsiveness in UC (Tang et al., 2025). A direct causal relationship between specific microbial taxa and UC pathogenesis has not yet been conclusively established. However, microbiome studies have consistently identified characteristic patterns of gut dysbiosis in patients with UC. These commonly include depletion of short chain fatty acids (SCFAs) producing bacteria, such as *Faecalibacterium prausnitzii*, *Roseburia intestinalis*, *Eubacterium rectale*, *Bifidobacterium longum*, and *Akkermansia muciniphila*, together with expansion of facultative anaerobes and potentially pro-inflammatory microorganisms, including *Escherichia, Shigella*, *Streptococcus*, *Veillonella*, and other members of the *Pseudomonadota (Proteobacteria)* phylum (Zhu et al., 2022; Świrkosz et al., 2023). Furthermore, it was shown that the composition of the mucosa-associated microbiota appears to reflect disease activity and clinical course in UC, suggesting its potential utility as a prognostic biomarker (Nishihara et al., 2021). Taken together, the composition and metabolic activity of the gut microbiota should be considered an important determinant of therapeutic outcomes in UC, as growing evidence indicates that microbial communities may influence disease activity, clinical course, and response to pharmacological interventions.

While considerable advances have been achieved in disease management, no curative treatment targeting the underlying cause of UC is currently available. Therapeutic management is tailored according to disease severity and extent, with treatment options including aminosalicylates (5-ASAs), corticosteroids, immunomodulators, biologic agents, and, more recently, small-molecule therapies (D’Amico et al., 2024). Aminosalicylates, particularly mesalazine, remain the cornerstone of treatment for mild-to-moderate disease, whereas corticosteroids are primarily used for induction of remission during active flares. Patients with moderate-to-severe UC may require escalation to biologic therapies targeting key inflammatory pathways, including TNF, integrins, or interleukin signaling. Antibiotics are not routinely recommended for UC management, although they may be prescribed in selected clinical situations, including infectious complications, pouchitis, or microbiota-targeted interventions (Noviello et al., 2025; Ferretti et al., 2022). A considerable proportion of patients fail to achieve sustained remission, experience loss of response over time, or require treatment escalation due to inadequate efficacy. Moreover, the long-term use of certain therapies is associated with adverse effects, while biologics and small-molecule therapies may be limited by primary or secondary non-response, immunogenicity, and high treatment costs (Singh et al., 2023). Consequently, there is growing interest in identifying complementary therapeutic approaches capable of improving treatment efficacy, prolonging remission, reducing disease recurrence, and supporting a more personalized management of UC to address the substantial interindividual variability in therapeutic response.

Given the close association between dysregulated inflammatory signaling and gut dysbiosis in UC, restoring immune homeostasis and microbial balance has emerged as an important therapeutic goal. Consequently, therapeutic interventions used in UC not only target inflammatory pathways but may also influence the composition and metabolic activity of the gut microbiota (Bartocci et al., 2026). Conversely, accumulating evidence suggests that the gut microbiome contributes to interindividual variability in treatment response through the modulation of host immunity, microbial metabolism, and epithelial barrier function (Wilmanski et al., 2022; Lei et al., 2025; Bartocci et al., 2026). These reciprocal interactions between pharmacological therapies and the gut microbiota have given rise to the field of pharmacomicrobiomics (Gronich et al., 2025), which investigates how drugs shape microbial communities and how the microbiota, in turn, influences drug efficacy, safety, and therapeutic outcomes. Several microbiota-directed approaches, including fecal microbiota transplantation, probiotics, and prebiotics, have been explored as adjunctive strategies for the management of IBD. Among these interventions, probiotics have been investigated most extensively and increasing attention has been focused on the implementation of probiotics as adjunctive therapeutic strategies in UC, as summarized by several recent reviews (Guo et al., 2024; Xu et al., 2025; Niu et al., 2026). Reported benefits of probiotics include modulation of gut microbial composition and metabolic activity, restoration of epithelial barrier integrity, regulation of mucosal immune responses, attenuation of pro-inflammatory signaling pathways and may additionally exert antioxidant effects (El-Saadony et al., 2026; Guo and Lv, 2023; Vallejos et al., 2025; Caliman-Sturdza et al., 2026). Importantly, probiotic efficacy in UC cannot be generalized across all commercially available preparations, as clinical benefits are highly strain- and formulation-dependent. To date, the most consistent evidence supports the use of *Escherichia coli* Nissle 1917 (Mutaflor®) and the multi-strain probiotic preparations the De Simone formulation (Visbiome® or Vivomixx®) and Bifid Triple Viable Capsules, which have demonstrated efficacy in both the induction and maintenance of remission in patients with mild-to-moderate UC (Kruis et al., 2004; Bibiloni et al., 2005; Sood et al., 2009; Oh et al., 2021; Tian et al., 2020). Beyond their established adjunctive use in UC, probiotics are particularly relevant in the context of drug interactions because viable, metabolically active microorganisms may influence pharmacotherapy both indirectly through modulation of the intestinal ecosystem and host responses, and directly through strain-specific metabolic activities, including drug biotransformation and bioaccumulation. These characteristics provide a strong rationale for examining probiotics within the framework of microbiota-drug interactions.

Although probiotics have attracted increasing attention as a therapeutic adjunct in UC, their potential interactions with concomitant pharmacological therapies remain insufficiently explored (Zhao et al., 2023). Accumulating evidence from non-IBD settings suggests that probiotic administration can modify both the pharmacokinetic and pharmacodynamic properties of selected drugs (Purdel et al., 2023; Matuskova et al., 2014; Kim et al., 2018; Matuskova et al., 2017; Saputri et al., 2018). Experimental studies have demonstrated that these interactions may alter the pharmacokinetic profile of orally administered drugs in a strain- and drug-specific manner. For example, administration of *Escherichia coli* Nissle 1917 or *Lacticaseibacillus casei* increased systemic exposure to the antiarrhythmic drug amiodarone in rats (Matuskova et al., 2014; Matuskova et al., 2017), whereas *Limosilactobacillus reuteri* K8, but not *L. reuteri* K9, reduced the area under the curve (AUC) of orally administered acetaminophen in mice (Kim et al., 2018). In addition, probiotic supplementation has also been shown to enhance the therapeutic efficacy of antihypertensive treatment without detectable alterations in drug pharmacokinetics (Ramadhani and Lestari, 2025). Nevertheless, despite the central role of the gut microbiota in disease pathogenesis and therapeutic responsiveness, comparable evidence in UC remains limited and fragmented. This knowledge gap is particularly important because probiotics are frequently used as adjunctive interventions in UC to support remission, restore intestinal homeostasis, and improve treatment outcomes. However, the extent to which probiotic supplementation influences the efficacy of conventional UC therapies through pharmacokinetic, pharmacodynamic, or microbiota-mediated mechanisms remains poorly understood. A deeper understanding of these interactions may help explain interindividual variability in therapeutic response and facilitate the development of microbiota-guided treatment strategies aimed at optimizing efficacy and achieving sustained disease control. Therefore, this review provides an up-to-date overview of probiotic-drug interactions in UC, with particular emphasis on microbiota- and metabolite-mediated mechanisms that may influence therapeutic efficacy. To our knowledge, this review is the first to synthesize available evidence on probiotic-drug interactions across the major therapeutic classes used in UC and to translate these findings into a drug-specific framework for probiotic use in UC management. Finally, we highlight key knowledge gaps and future research directions in this rapidly evolving field.

## 2 Mechanisms underlying microbiota-drug interactions relevant for UC

Interactions between the gut microbiota and pharmacological therapies can significantly contribute to interindividual variability in treatment response. These interactions may occur through both pharmacokinetic and pharmacodynamic mechanisms, influencing drug activation, metabolism, bioavailability, efficacy, and toxicity (Verdegaal and Goodman, 2024; Al-Btoosh et al., 2026). As summarized in Figure 1, microbiota-drug interactions can be broadly classified into three interconnected mechanisms: (i) direct microbial drug biotransformation, (ii) indirect modulation of host pharmacokinetic processes, and (iii) microbiota-mediated pharmacodynamic interactions. These pathways may also be modified by microbiota-directed interventions, including probiotics and postbiotics. Such interactions are particularly relevant in UC, where therapeutic outcomes are shaped not only by host factors but also by the gut microbial ecosystem and its metabolic activity.

**Figure 1:**
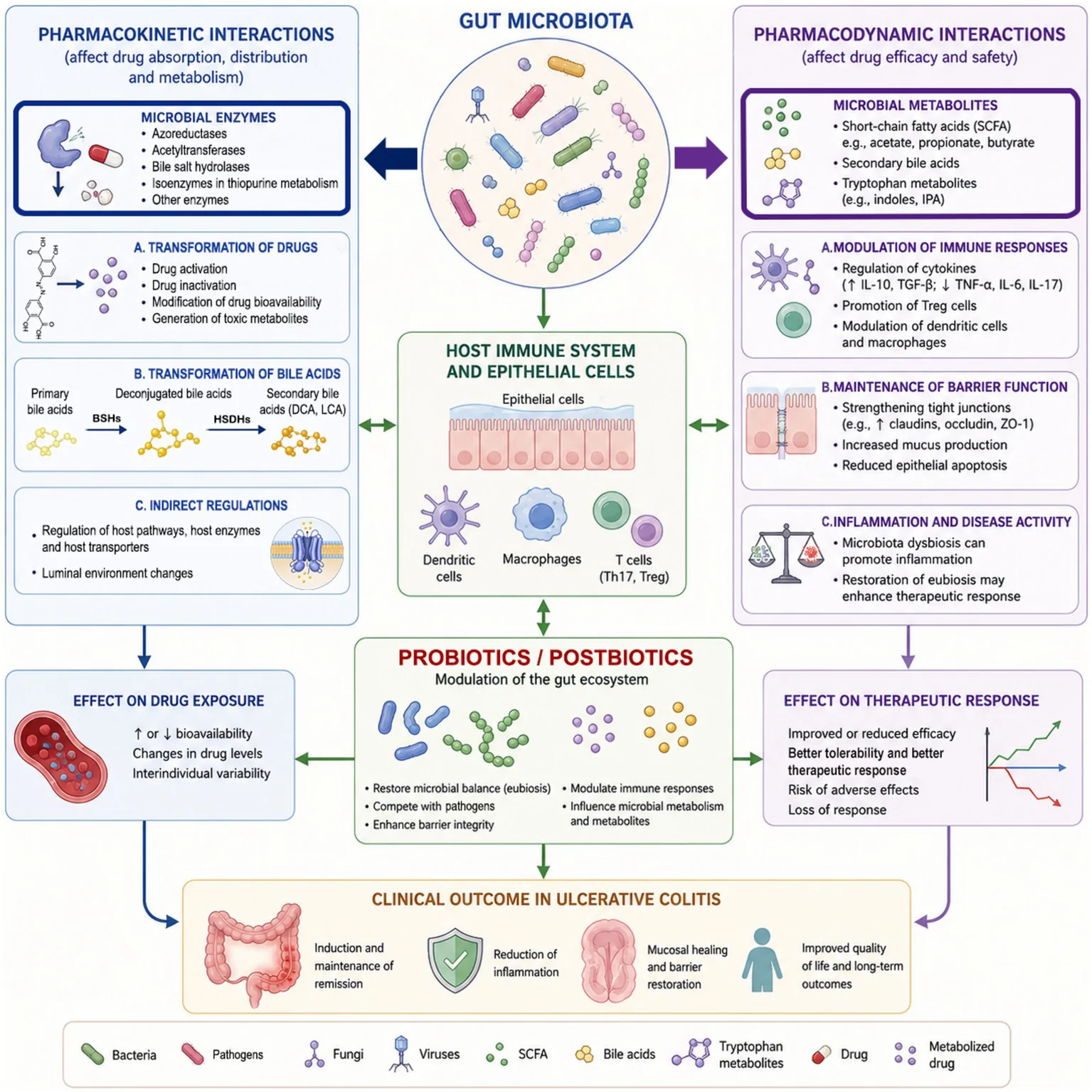
Mechanisms underlying microbiota–drug interactions and potential sites of action of probiotics and postbiotics in ulcerative colitis. Gut microbiota influences the pharmacokinetics and pharmacodynamics of ulcerative colitis therapies through three interconnected mechanisms: (i) Direct microbial drug biotransformation, (ii) indirect modulation of host pharmacokinetic processes, including drug-metabolizing enzymes, transporters, epithelial barrier integrity, and intestinal permeability, and (iii) microbiota-mediated pharmacodynamic interactions driven by microbial metabolites and immune signaling. Microbiota-directed interventions, including probiotics and postbiotics, may modulate each of these pathways by reshaping microbial composition and metabolic activity, thereby influencing drug exposure, therapeutic efficacy, and clinical outcomes in ulcerative colitis. BSH, bile salt hydrolase; DCA, deoxycholic acid; HSDH, hydroxysteroid dehydrogenase; IPA, indole-3-propionic acid; LCA, lithocholic acid; SCFAs, short-chain fatty acids; ZO-1, zonula occludens-1. Arrows indicate the direction of change: ↑, Increase; ↓, Decrease.

### 2.1 Direct microbiota biotransformation of drugs

One of the best-characterized mechanisms involves direct microbial biotransformation of drugs. Gut microorganisms harbor a diverse repertoire of unique enzymes that shape host metabolic health, as well as microbial isozymes that can mimic or complement host enzymatic functions, contributing to the metabolism of signaling molecules, nutrients, and xenobiotics (Wilson and Nicholson, 2017; Ding et al., 2026; Collins and Patterson, 2020).

Among the most clinically relevant examples of microbial enzymes involved in UC pharmacotherapy are azoreductases, which mediate the microbiota-dependent activation of aminosalicylate prodrugs such as sulfasalazine or olsalazine. These bacterial enzymes cleave azo bonds, releasing the active compound mesalazine directly within the colon (Sousa et al., 2014). Azoreductase activity has been primarily described in anaerobic bacteria belonging to the genera *Clostridium* and *Eubacterium* (Chen et al., 2004; Morrison and John, 2015). Interestingly, azoreductase activity has also been reported in selected probiotic strains, including *Lactobacillus acidophilus*, *Bifidobacterium animalis* subsp. *lactis*, and *Streptococcus salivarius*, but this activity appears to be highly strain-dependent (Lee et al., 2012). The therapeutic potential of exploiting microbial azoreductase activity has recently been demonstrated using a colon-targeted probiotic-drug co-delivery system, in which sulfasalazine and *Clostridium butyricum* spores possessing intrinsic azoreductase activity were co-encapsulated within a drug delivery system. Following colonic release, germination of the spores enhanced microbial conversion of sulfasalazine to 5-aminosalicylic acid while simultaneously restoring gut microbiota homeostasis and epithelial barrier function, resulting in superior therapeutic efficacy in experimental colitis (Zhao et al., 2025). These findings illustrate that the gut microbiota represents not only a passive determinant of drug metabolism but also a potential therapeutic target that can be intentionally engineered to optimize prodrug activation and improve treatment efficacy.

Furthermore, the gut microbiota harbors homologues of several host enzymes involved in thiopurine metabolism, including thiopurine S-methyltransferase (TPMT)-like methyltransferases, glutathione S-transferases, and xanthine oxidase/xanthine dehydrogenase-like enzymes, which may contribute to the metabolism of immunomodulatory drugs such as azathioprine and thioguanine (Atreya and Neurath, 2017; Lazarević et al., 2022). Bacterial TPMT homologues capable of methylating 6-mercaptopurine to the inactive metabolite 6-methylmercaptopurine have been identified in several *Pseudomonas* species (Scheuermann et al., 2003).

Another important mechanism is bile acid metabolism, which provides a functional link between the gut microbiota, host metabolism, and drug response. Through the activity of microbial bile salt hydrolases, conjugated primary bile acids are deconjugated and subsequently converted into secondary bile acids by additional microbial enzymes, including hydroxysteroid dehydrogenases. These microbial transformations generate a structurally diverse bile acid pool capable of regulating host physiology through bile acid receptors and xenobiotic-sensing pathways, including farnesoid X receptor (FXR), Takeda G protein-coupled receptor 5 (TGR5), and pregnane X receptor (Gadaleta et al., 2022; Bourgin et al., 2021; Labbé et al., 2014; Zhang et al., 2025).

Collectively, these findings demonstrate that gut microbial enzymatic activity can directly influence the metabolism and bioavailability of UC medications, thereby affecting their therapeutic efficacy. Elucidation of these microbial drug-metabolizing pathways may enable the identification of microbiome-based biomarkers for predicting treatment response and the development of personalized therapeutic strategies (Guthrie and Kelly, 2019; Neelam et al., 2026).

### 2.2 Indirect modulation of host metabolism

In addition to microbial enzymes, the commensal gut microbiota generates a remarkably diverse array of bioactive metabolites, including SCFAs, secondary bile acids, tryptophan-derived metabolites, bacteriocins, essential vitamins, phenolic compounds and bacterial extracellular vesicles (BEVs), which participate in host metabolism, immune regulation and microbial homeostasis (Xue et al., 2025; Russo et al., 2019). These microbial metabolites also influence drug disposition indirectly by modulating host metabolic pathways. Secondary bile acids were shown to regulate xenobiotic-sensing pathways involving pregnane X receptor and constitutive androstane receptor, thereby influencing the expression and activity of drug-metabolizing enzymes and membrane transporters (Jourová et al., 2020). Members of the cytochrome P450 (CYP) family, particularly CYP3A4, together with the efflux transporter P-glycoprotein (ABCB1) represent very well characterized host factors affected by microbiota-dependent signaling (Foley et al., 2022; Degraeve et al., 2023; Wang et al., 2023a). Consistent with this concept, administration of a compound probiotic formulation altered the pharmacokinetics of several CYP probe substrates in rats, suggesting that selected probiotics may modulate the activity of specific CYP isoenzymes involved in host drug metabolism (Zhang et al., 2024a, 2024b). In addition, microbiota-induced alterations in epithelial barrier integrity, mucus thickness, and intestinal permeability may further influence the absorption, bioavailability, and systemic exposure of orally administered drugs, whereas changes in luminal pH may additionally affect the dissolution and release of pH-dependent formulations (Zhang et al., 2021; Vinarov et al., 2021; Moutaharrik et al., 2023). The importance of these host-mediated mechanisms is further supported by computational pharmacokinetic modeling, which demonstrated that incorporating microbiota-dependent regulation of intestinal CYP enzymes and drug transporters significantly improved prediction of tacrolimus and sulfasalazine pharmacokinetics (Cooper and Bhushan, 2024). These findings highlight that microbiota-drug interactions extend beyond direct microbial biotransformation and also involve modulation of host metabolic pathways.

### 2.3 Pharmacodynamic interactions mediated by microbial metabolites

Alongside regulating host drug metabolism, microbiota-derived metabolites also contribute to pharmacodynamic interactions by modulating epithelial barrier integrity, mucosal immunity, and inflammatory signaling. SCFAs, secondary bile acids, and tryptophan-derived metabolites regulate key pathways involved in UC pathogenesis, including NF-κB, JAK/STAT, peroxisome proliferator-activated receptor *γ* (PPAR-*γ*), FXR, and TGR5 signaling, thereby influencing therapeutic responses independently of changes in systemic drug exposure (Shen et al., 2025; Liang et al., 2026).

Among SCFA-producing commensals, *Faecalibacterium* and *Roseburia* represent the principal butyrate-producing genera and contribute to intestinal homeostasis through butyrate-mediated epigenetic regulation, reinforcement of epithelial barrier integrity, and immunometabolic modulation (Zhou et al., 2018; Deng et al., 2025). The anti-inflammatory effects of SCFAs in UC have been extensively characterized, whereas considerably less is known about their direct interactions with pharmacological therapies. Current evidence suggests that SCFAs primarily complement conventional treatment by maintaining intestinal homeostasis, regulating epithelial metabolism, modifying host metabolic enzyme expression, and shaping gut microbial composition rather than directly altering drug pharmacokinetics (Vagnerová et al., 2024; Akhtar et al., 2021).

Microbial metabolites derived from tryptophan are important regulators of mucosal immunity through activation of the aryl hydrocarbon receptor (AhR). Key indol derivates, including indole-3-aldehyde, indole-3-acetic acid, indole-3-propionic acid, and indole acrylic acid promote IL-22 production, strengthen the epithelial barrier integrity, and regulate the balance between Treg and Th17 lymphocytes, thereby contributing to intestinal immune homeostasis (Li, 2023a; Li et al., 2025; Rondeau et al., 2026). Direct interactions between tryptophan metabolites and UC pharmacokinetics have not been demonstrated. Nevertheless, these metabolites may complement the pharmacodynamic effects of mesalazine, corticosteroids, biologics, and JAK inhibitors by acting on overlapping immunoregulatory pathways, including AhR, NF-κB, and JAK/STAT signaling (Wang et al., 2023b; Sun et al., 2026; Gao et al., 2026). Together with SCFAs and secondary bile acids, tryptophan-derived metabolites further illustrate that restoration of microbial metabolic function, rather than taxonomic composition alone, may substantially contribute to therapeutic response in UC.

Accordingly, interactions between microbiota and drugs in UC involve multiple interconnected mechanisms including direct microbial biotransformation of drugs, regulation of host drug metabolism, and microbiota-mediated pharmacodynamic modulation of intestinal homeostasis. Understanding these mechanisms provides a conceptual framework for interpreting how conventional UC therapies interact with the intestinal ecosystem and how microbiota-directed interventions, including probiotics and postbiotics, may ultimately optimize therapeutic responses.

## 3 Drugs used in UC and their interactions with gut microbiota and probiotics

The impact of IBD therapies on the gut microbiota, and vice versa, has recently attracted considerable attention within the field of pharmacomicrobiomics (Lazarević et al., 2025; Yao et al., 2025; Bartocci et al., 2026; Collins et al., 2026). Accumulating evidence indicates that therapeutic agents used in UC can induce profound microbial and metabolic alterations. Understanding these therapy-associated microbial changes is essential, as they may contribute to treatment efficacy, interindividual variability in therapeutic response, and the success of subsequent microbiota-targeted interventions. The following sections summarize the major therapeutic classes used in UC and discuss their potential interactions with the gut microbiota and probiotics, with particular emphasis on microbiota-mediated and metabolite-driven mechanisms that may influence treatment response. Table 1 presents the major available experimental and clinical evidence for pharmacokinetic and pharmacodynamic probiotic–drug interactions across these therapeutic classes.

**Table 1:** Experimental and clinical evidence of pharmacokinetic (A) and pharmacodynamic (B) interactions between probiotics and UC therapies.

UC therapy	Drug	Probiotic intervention	Level of evidence	Key pharmacokinetic findings	Representative studies
Part A: probiotic–drug pharmacokinetic interactions in UC					
aminosalicylates	Olsalazine	*Lactobacillus acidophilus*	Preclinical study (TNBS colitis, rats)	no significant pharmacokinetic effects on olsalazine, 5-ASA, and N-Ac-5-ASA; no alterations in azoR/NAT-related microbiota or metabolic enzymes	Li et al. (2022)
	Sulfasalazine	*Clostridium butyricum* spores (co-delivery system)	Preclinical study (DSS colitis, mice)	enhanced microbial activation of sulfasalazine to 5-ASA	Zhao et al. (2025)
	Olsalazine	thiolated *Clostridium butyricum* (co-delivery system)	Preclinical study (*in vitro*, DSS- and TNBS colitis, mice)	↑ microbial azoreductase activity ↑ enhanced olsalazine conversion to 5-ASA	Song et al. (2026)
Immunomodulators	Azathioprine	*Lactobacillus helveticus, Lactobacillus acidophilus, Lacticaseibacillus rhamnosus,* *Bifidobacterium longum* + deoxycholate	*In vitro study* (PAMPA model)	↑ Passive permeability of AZA; potential effects on active drug transporters could not be confirmed using the applied method; ↓ AZA levels in the presence of probiotics, suggesting bacterial bioaccumulation and/or enzymatic biotransformation	Danić et al. (2025)
	Tacrolimus Everolimus Cyclosporin A	*Bacillus subtilis TO-A*, *Clostridium butyricum* *(TO-A and MIYAIRI588)*	*In vitro* study Preclinical study (healthy mice)	Promoted *in vitro* degradation of tacrolimus and everolimus, ↓ residual concentrations; no effect on cyclosporine A degradation *B. subtilis* ↓ tacrolimus peak blood concentration (C < sub > max</sub>) and systemic exposure	Tsuchiya et al. (2025)
Part B: probiotic–drug pharmacodynamic interactions in UC					
Aminosalicylates	Olsalazine	*Lactobacillus acidophilus*	Preclinical study (TNBS colitis, rats)	improved therapeutic efficacy was associated with microbiota modulation	Li et al. (2022)
	Mesalazine	Bifida Triple Viable Capsules *(Bifidobacterium longum, Lactobacillus acidophilus, Enterococcus faecalis)*	Retrospective clinical study (patients with IBD)	↓ systemic inflammatory and immunological markers (IgA, IgG, IgM, TNF-α, IL-6, CRP); improved clinical and endoscopic disease activity index	Tan et al. (2022)
	Mesalazine	*Lacticaseibacillus rhamnosus GG* + vit D3	Randomized double-blind placebo-controlled clinical trial (patients with UC)	No improvement in short-term clinical outcomes compared with placebo; ↓Fecal calprotectin, ↑Frequency of circulating IL-12-producing CD4 + T cells; favorable microbiota remodeling with enrichment of SCFA-producing taxa (↑ *Coprococcus* and *Fusicatenibacter*) ↑α-diversity in responders	Maragno et al. (2026)
	Mesalazine	*Lacticaseibacillus casei*	Preclinical study (acetic acid induced colitis, mice)	↓Macroscopic and microscopic intestinal damage; ↓DAI; ↓Inflammatory mediators (NO, MPO, IL-1β, IL-6, and TNF-α); downregulated expression of IL-1β, IL-6, TNF-α, iNOS, and COX-2; demonstrated synergistic effects with mesalazine	Bahrami et al. (2024)
	Mesalazine	*Escherichia coli* Nissle 1917	Prospective observational clinical study (patients with UC)	improved maintenance of clinical remission in patients with UC	Oh et al. (2021)
	Mesalazine	*Bifidobacterium* triple viable capsules	Meta-analysis (13 randomized controlled clinical trials; patients with UC)	↓DAI and enhanced mucosal healing; improved clinical efficacy with combination therapy; benefits were observed when probiotics were administered for at least 8 weeks	Tian et al. (2020)
	Sulfasalazine	*Clostridium butyricum spores (co-delivery system)*	Preclinical study (DSS colitis, mice)	Restored gut microbiota (↑microbial richness/diversity, ↑ *Lactobacillaceae*, *Lachnospiraceae*, *Akkermansiaceae*, and *Clostridia*); improved barrier integrity (↑ZO-1, occludin, claudin-1); ↓intestinal inflammation (↓IL-6, IL-1β, TNF-α, TLR4, NF-κB p65; ↑IL-10)	Zhao et al. (2025)
	Olsalazine	thiolated *Clostridium butyricum* (co-delivery system)	Preclinical study (DSS and TNBS-colitis, mice)	↑Butyrate production; improved disease activity ↓ DAI, attenuated body weight loss ↓Intestinal inflammation and epithelial injury(↑ MUC2, ZO-1, and occludin) Gut microbiota modulation (↑ α-diversity; ↑ Bacteroidota, Bacillota, and Verrucomicrobiota; ↓ Pseudomonadota)	Song et al. (2026)
	Mesalazine	*Lactiplantibacillus plantarum* AS21	Preclinical study (DSS colitis, mice)	Time-dependent enhancement of mesalazine efficacy; ↓DAI and body weight loss; improved mucosal architecture and barrier integrity (↑claudin-1, occludin); ↓Pro-inflammatory cytokines (TNF-α, IL-6, IL-1β, IFN-*γ*); ↑Antioxidant defenses (CAT, SOD, GSH); partial restoration of gut microbial diversity; modulation of glycerophospholipid and steroid metabolic pathways.	Bacha et al. (2026)
Corticosteroids	Prednisone	De Simone Formulation	Randomized double-blind clinical trial (dogs)	no additional protection against glucocorticoid-induced gastric bleeding or mucosal injury compared with prednisone alone	Rak et al. (2023)
	Tacrolimus	*Lactiplantibacillus plantarum* 550	Preclinical study (DSS colitis, mice)	modulated gut microbiota and bile acid metabolism via downregulation of the bile acid receptors TGR5 and FXR and ↑ cecal TCDCAlevels; downregulation of IFN-*γ* and IFN-α responses and IL-2–STAT5 signaling	Lv et al. (2023)
	Cyclosporine A + syndecan	mixture of *Lacticaseibacillus rhamnosus, Saccharomyces boulardii, Bifidobacterium breve, Bifidobacterium animalis* subsp. *lactis, Lactobacillus acidophilus, Lactiplantibacillus plantarum Limosilactobacillus reuteri*	Preclinical study (DSS colitis, mice)	no improvement in clinical signs compared with treatment alone	Dosh et al. (2024)
Biologics	Infliximab	*Bacteroides thetaiotaomicron*	Preclinical study (DSS colitis, mice)	enhanced epithelial barrier-associated gene expression (↑*Ocln*, ↑*Tff3,* ↑*Muc2*; ↓*Ace2*) synergistically ↓ colitis severity	Badi et al. (2025)
	Infliximab	*Bifidobacterium longum* CECT 7894	Preclinical study (DSS colitis, mice)	↓ DAI, improved histopathological score; restored intestinal barrier integrity through ↑ TJP expression; enriched beneficial taxa ↑*Bifidobacterium* and *Blautia*; ↑Production of anti-inflammatory secondary bile acids.	Xiao et al. (2022)
	Infliximab	*Lactobacillus gasseri*	Preclinical study (DSS colitis, mice)	↓Expression of pro-inflammatory cytokines (TNF-α, IFN-*γ*, IL-6, and IL-17A); ↑IL-10 production and improved epithelial barrier function; enrichment of *Prevotella* spp.	Han et al. (2022)

5-ASA, 5-aminosalicylic acid; AZA, azathioprine; Cmax, maximum plasma concentration; COX-2, cyclooxygenase-2; CRP, C-reactive protein; DAI, disease activity index; DSS, dextran sulfate sodium; FXR, farnesoid X receptor; IBD, inflammatory bowel disease; IFN, interferon; IgA, immunoglobulin A; IgG, immunoglobulin G; IgM, immunoglobulin M; IL, interleukin; iNOS, inducible nitric oxide synthase; MPO, myeloperoxidase; NAT, N-acetyltransferase; NF-κB, nuclear factor kappa B; NO, nitric oxide; PAMPA, parallel artificial membrane permeability assay; RCT, randomized controlled trial; SCFA, short-chain fatty acid; TCDCA, taurochenodeoxycholic acid; TGR5, Takeda G-protein-coupled bile acid receptor 1; TJP, tightjunction protein; TNBS, 2,4,6-trinitrobenzenesulfonic acid; TNF-α, tumor necrosis factor-alpha; UC, ulcerative colitis; ZO-1, zonula occludens-1; CAT, catalase; SOD, superoxide dismutase, GSH, glutathione. Arrows indicate the direction of change: ↑, increased; ↓, decreased.

### 3.1 Aminosalicylates

Aminosalicylates, including mesalazine, sulfasalazine, balsalazide, and olsalazine, have represented the cornerstone of therapy for mild-to-moderately active UC for several decades. These agents are well established as effective, safe, and cost-efficient therapeutic options for both the induction and maintenance of remission (Murray et al., 2020). Among mentioned drugs, mesalazine is currently the most widely prescribed 5-ASA formulation due to its favorable safety profile and broad clinical applicability (Raine et al., 2022). Owing to its predominantly local mode of action within the intestinal mucosa, mesalazine is available in a variety of oral and topical formulations designed to optimize drug delivery according to disease location and extent. Mesalazine exerts its therapeutic effects primarily through local anti-inflammatory activity within the colonic mucosa. Despite decades of clinical use and extensive investigation, its exact mechanism of action remains incompletely understood (Veloso et al., 2021). Nevertheless, 5-ASA is known to inhibit key inflammatory pathways, including NF-κB signaling, pro-inflammatory cytokine production (TNF-α, IL-6), and cyclooxygenase and lipoxygenase-mediated eicosanoid synthesis (Berends et al., 2019; Xu et al., 2014; Kaiser et al., 1999). In addition, mesalazine acts as an antioxidant and promotes epithelial barrier integrity by enhancing the expression of several tight-junction proteins (JAM-A, occludin, claudins, E-cadherin) and supporting mucosal healing (Khare et al., 2019; Wang et al., 2025). A major advance in understanding its mode of action was the identification of PPAR-*γ* as a molecular target of mesalazine (Rousseaux et al., 2005). Mesalazine acts as a PPAR-*γ* agonist, thereby suppressing pro-inflammatory signaling pathways while promoting epithelial barrier function and mitochondrial oxygen consumption in colonocytes, which helps maintain epithelial hypoxia and limits the expansion of facultative anaerobic pro-inflammatory *Enterobacterales*, including *Escherichia coli* (Cevallos et al., 2021). Beyond these direct effects on the host, accumulating evidence suggests that mesalazine may also influence the composition and metabolic activity of the gut microbiota, thereby contributing to restoration of intestinal homeostasis and potentially affecting treatment efficacy and patient response (Becker et al., 2023).

#### 3.1.1 Interactions between 5-ASAs and gut microbiota

Among UC therapies, mesalazine has been one of the most extensively studied in the context of microbiota-drug interactions, as highlighted by several reviews (Radhakrishnan et al., 2025; Becker et al., 2023; Lazarević et al., 2025; Bartocci et al., 2026). Accumulating clinical evidence indicates that mesalazine consistently influences both microbial composition and mucosal homeostasis (Mehta et al., 2023). Initial evidence from mucosal biopsy analyses in patients with IBD demonstrated that mesalazine treatment was associated with reduced mucosal bacterial density, decreased bacterial adherence to the epithelial surface, and increased mucus thickness, suggesting improved barrier function and reduced colonization of the mucosa by invasive pathogens (Swidsinski et al., 2007). Subsequent studies demonstrated that higher mucosal concentrations of 5-ASA are associated with increased bacterial diversity and enrichment of taxa generally linked to intestinal health, including *Faecalibacterium (prausnitzii), Blautia, Bacteroides, Coprococcus, Roseburia, Ruminococcus, Oscillospira*, *Bifidobacterium* and *Odoribacter*, together with a reduction in potentially pro-inflammatory taxa such as *Pseudomonadota* members, including *Escherichia–Shigella* and *Klebsiella.* Interestingly, responses within *Bacteroidota*, particularly among *Prevotella*-associated taxa, vary across studies following mesalazine treatment, with both enrichment and depletion reported (Olaisen et al., 2019; Xu et al., 2018; Wang et al., 2021; Maragno et al., 2026). This variability likely reflects marked heterogeneity of this dominant phylum, which includes taxa with divergent biological roles, including both associated with dysbiosis and inflammation and anti-inflammatory members. Although inconsistencies exist at lower taxonomic levels, studies consistently report an enrichment of SCFA-producing commensals following mesalazine therapy. This functional microbial shift parallels the established anti-inflammatory effects of mesalazine and may contribute to restoration of intestinal homeostasis.

Using murine experiments, Wada et al. (2023) provided causal evidence that mesalazine directly induces changes in the gut microbiota that, in turn, shape host mucosal immune status. In a vertical transmission model, they demonstrated that 5-ASA-modified microbiota, transferred from mothers to offspring in the absence of direct drug exposure, was associated with a shift in host mucosal immunity toward an anti-inflammatory phenotype and reduced susceptibility to DSS-induced colitis. This finding was further supported by another study showing that, following antibiotic depletion of the endogenous microbiota, mesalazine lost its ability to alleviate colitis and restore the intestinal barrier (Wang et al., 2025). These results indicate that the beneficial effect of 5-ASA is at least partially microbiota-dependent and that the effects on the barrier, immunity and microbiome are functionally linked.

Recent studies further suggest that baseline microbiota composition may influence therapeutic response. Patients responding to mesalazine tend to exhibit lower abundances of *Pseudomonadota* and higher abundances of SCFA-producing taxa, whereas treatment failure has been associated with depletion of *Faecalibacterium prausnitzii*, *Blautia massiliensis*, and *Phascolarctobacterium faecium*, together with enrichment of *Klebsiella pneumoniae* and other dysbiosis-associated taxa (Schierová et al., 2020; Dang et al., 2025; Xu et al., 2018; Wang et al., 2021). While these studies primarily identified taxonomic signatures associated with treatment response, emerging pharmacomicrobiomic evidence suggests that the functional metabolic capacity of the gut microbiota may represent an equally important determinant of mesalazine efficacy. As discussed in the section on microbiota-drug interaction mechanisms, microbial activation of sulfasalazine and olsalazine by bacterial azoreductases is well established. In contrast, mesalazine has traditionally been considered independent of microbiota-mediated activation because it is administered in its pharmacologically active form. However, recent pharmacomicrobiomic studies challenge this assumption by demonstrating that gut microbial enzymes can directly metabolize active 5-ASA. Mehta et al. (2023) identified twelve previously uncharacterized gut microbial acetyltransferases capable of converting mesalazine into the clinically inactive metabolite N-acetyl-5-ASA. Four of these enzymes (three thiolases and one acyl-CoA *N*-acyltransferase) were prospectively associated with mesalazine treatment failure, and the presence of three or more corresponding microbial genes increased the risk of treatment failure by approximately threefold. This association was subsequently validated in an independent patient cohort. From a clinical perspective, these findings indicate that taxonomic identification alone may not fully capture variability in the metabolic activity of the gut microbiota, including its capacity to metabolize and inactivate 5-ASA, which may directly contribute to interindividual differences in treatment response. Collectively, these observations suggest that the gut microbiota influences mesalazine efficacy not only through differences in microbial composition but also through its functional metabolic capacity. While specific microbial signatures may serve as biomarkers of treatment response, emerging pharmacomicrobiomic evidence indicates that functional microbiome profiling may provide a more informative framework for predicting mesalazine responsiveness and supporting microbiome-guided therapeutic strategies.

#### 3.1.2 Interplay between probiotics and 5-ASAs

Given the complementary mechanisms through which mesalazine and probiotics modulate the intestinal microenvironment, their combined administration has attracted considerable interest as a strategy to further improve therapeutic outcomes in UC (Niu et al., 2026; Ma et al., 2024; Xu et al., 2025). Selected probiotic formulations, particularly *Escherichia coli* Nissle 1917 (Mutaflor®) and the multi-strain the De Simone formulation, have demonstrated efficacy comparable to 5-ASA in inducing and maintaining remission in patients with mild-to-moderate UC (Kruis et al., 2004; Bibiloni et al., 2005; Sood et al., 2009; Oh et al., 2021). Consequently, several clinical studies have evaluated combined administration of probiotics and mesalazine, demonstrating that this strategy is safe and may improve clinical remission rates, prolong remission, and reduce intestinal inflammation compared with mesalazine monotherapy, particularly in patients with mild-to-moderate UC (Tian et al., 2020; Maragno et al., 2026). However, robust evidence demonstrating the superiority of probiotic-mesalazine combination therapy over mesalazine alone remains limited, with most mechanistic and efficacy data originating from experimental studies on UC animal models and relatively few randomized clinical trials (El-Baz et al., 2020; Mardini and Grigorian, 2014; Oh et al., 2021; Bahrami et al., 2024). Furthermore, the therapeutic efficacy of adjunctive probiotic supplementation appears to depend on multiple factors, including probiotic strain specificity, formulation, dosage regimen, treatment duration, and disease activity at the time of administration (Sarita et al., 2025). Consistent with this concept, Bacha et al. (2026) demonstrated in DSS-induced murine colitis model that the adjunctive effects of *Lactiplantibacillus plantarum* AS21 on mesalazine therapy increased over time, with more pronounced improvements in clinical outcomes, gut microbial composition, and immunometabolic parameters observed during the second and third weeks of intervention.

Available evidence regarding pharmacokinetic interactions between probiotics and 5-ASA-based therapy is limited to a single study by Li et al. (2022), who investigated the effect of *Lactobacillus acidophilus* on olsalazine metabolism in a trinitrobenzenesulfonic acid (TNBS)-induced rat model of colitis. Pharmacokinetic analyses demonstrated that probiotic supplementation did not alter the systemic or colonic exposure to olsalazine, 5-ASA, or N-acetyl-5-ASA, nor did it affect microbial azoreductase- or N-acetyltransferase-mediated metabolism in the plasma and feces of rats with UC, indicating that the improved therapeutic efficacy of combination therapy was unlikely to result from altered 5-ASA pharmacokinetics. Instead, *L. acidophilus* appeared to enhance therapeutic efficacy through modulation of the gut microbiota and microbial metabolic pathways, including amino acid, phenolic acid, oligosaccharide, and peptidoglycan metabolism, without affecting drug disposition.

Accordingly, the meta-analysis by Tian et al. (2020) confirmed the clinical benefit of combining probiotics with 5-ASA therapy. The majority of studies included in the meta-analysis primarily evaluated complementary pharmacodynamic effects rather than clinically relevant alterations in drug exposure. Because probiotic effects are highly strain-specific, both strain selection and formulation composition appear to be critical determinants of therapeutic efficacy. Recent experimental evidence further indicates that rationally designed multi-strain probiotic consortia may be more effective than single strains by exploiting metabolic cross-feeding between lactate-producing and butyrate-producing bacteria. In an *in vitro* colonic microbiota model using fecal samples from patients with UC, co-administration of *Weizmannia coagulans* with the butyrate-producing bacteria *Anaerostipes caccae* or *Clostridium butyricum* significantly enhanced butyrate production, whereas administration of individual strains alone failed to restore butyrogenesis (Inokuma et al., 2025). Consistent with the potential therapeutic advantages of multi-strain formulations, adjunctive administration of the multi-strain probiotic formulation BifidoTriple (*Bifidobacterium longum*, *Lactobacillus acidophilus*, and *Enterococcus faecalis*) together with mesalazine significantly improved clinical and endoscopic disease activity scores while reducing systemic inflammatory markers and improving immune function compared with mesalazine monotherapy in patients with IBD (Tan et al., 2022). Similarly, supplementation with the multi-strain probiotic Symprove® (*Lacticaseibacillus rhamnosus* NCIMB 30174, *Lactiplantibacillus plantarum* NCIMB 30173, *Lactobacillus acidophilus* NCIMB 30175, and *Enterococcus faecium* NCIMB 30176) for 4 weeks significantly reduced intestinal inflammation compared with placebo in patients with quiescent or mildly active UC (Bjarnason et al., 2019).

Collectively, current evidence indicates that the efficacy of probiotic supplementation in combination with mesalazine depends not only on probiotic strain selection and formulation composition but also on treatment duration. Whereas short-term administration (approximately 7–10 days) may facilitate initial modulation of the gut microbiota, longer supplementation periods (3–24 months) have primarily been investigated for maintaining clinical remission and reducing the risk of relapse (Ma et al., 2024). Consistent with this, a meta-analysis by Peng et al. (2019) demonstrated that adjunctive probiotic therapy improved clinical remission rates during the first 4–8 weeks of treatment compared with 5-ASA monotherapy, although this advantage was no longer evident after 12 weeks. In contrast, Palumbo et al. (2016) reported sustained clinical improvement during 2 years of continuous probiotic supplementation combined with mesalazine. Overall, these findings support the concept that probiotics complement mesalazine predominantly through strain-specific pharmacodynamic mechanisms rather than pharmacokinetic interactions. These findings support the concept of precision probiotic therapy, in which strain selection is guided by complementary mechanisms of action rather than empirical supplementation. Taken together, the available evidence illustrates the bidirectional nature of mesalazine–microbiota interactions, in which mesalazine reshapes the gut microbiome while selected probiotic strains complement its therapeutic effects primarily through strain-specific pharmacodynamic mechanisms.

The concept of intentionally exploiting probiotic-mediated drug activation has recently been extended to probiotic-drug co-delivery systems. Zhao et al. (2025) developed a system designed to enhance the microbial activation of sulfasalazine, while Song et al. (2026) combined thiolated *Clostridium butyricum* with olsalazine to increase bacterial azoreductase activity and promote its conversion to 5-ASA. Importantly, enhanced olsalazine activation was accompanied by improved therapeutic outcomes in both DSS- and TNBS-induced colitis models, linking probiotic-mediated drug biotransformation with downstream pharmacodynamic benefits. Beyond direct microbial drug activation, recent studies have explored engineered probiotic-drug delivery platforms aimed at improving targeted drug delivery while simultaneously exploiting probiotic-mediated effects. Gu et al. (2026) developed a single-cell delivery platform combining *Lacticaseibacillus rhamnosus* GG with 5-ASA, enabling intestinal-targeted probiotic delivery and inflammation-responsive drug release while promoting restoration of the intestinal barrier and gut microbiota. A more complex multimodal strategy was recently proposed by Liu et al. (2026), who developed a sequential delivery system integrating *Clostridium butyricum*, 5-ASA, and the microbial metabolite indole-3-acetic acid (IAA). The platform was designed to deliver 5-ASA to the colon while exploiting IAA-mediated modulation of the gut-brain axis. In experimental colitis, this combined approach promoted intestinal barrier repair, M2 macrophage polarization, and restoration of Th17/Treg balance, while also alleviating anxiety- and depression-like behaviors and reducing inflammatory recurrence. Similarly, Jiang et al. (2026) developed a biohybrid probiotic–drug delivery system combining *Bifidobacterium longum* with 5-ASA to simultaneously enhance bacterial viability and enable inflammation-responsive drug release. In a DSS-induced murine model, this approach reduced colitis severity and helped restore mucosal immune and microbial homeostasis, although its clinical potential remains to be validated. Although these approaches remain preclinical, they illustrate the potential of integrating microbial functionality with targeted drug delivery to optimize 5-ASA-based therapy.

### 3.2 Corticosteroids

Corticosteroids (CSs) are first-line molecules for induction therapy in patients with moderate-to-severe UC due to their potent anti-inflammatory effects and rapid onset of action (Iizuka et al., 2025). However, their use is limited to short-term disease control, as prolonged exposure is associated with significant adverse effects and does not contribute to long-term maintenance of remission or achievement of mucosal healing (Feuerstein et al., 2025). Consequently, recent consensus recommendations emphasize minimizing CSs exposure through rapid tapering strategies and timely transition to steroid-sparing therapies (Hanžel et al., 2025). Prednisone, prednisolone, methylprednisolone, and budesonide are the most commonly used CSs in UC. Following administration, these agents are generally well absorbed and undergo extensive host-mediated metabolism, predominantly via CYP3A-dependent pathways (Möhlmann et al., 2024). Budesonide differs from conventional corticosteroids by undergoing substantial first-pass metabolism, by CYP3A4, leading to a low systemic bioavailability of 10–20% following oral administration, resulting in lower systemic exposure and an improved safety profile (Jönsson et al., 1995; Lee et al., 2024). In addition to host metabolism, drug transport mechanisms and disease-associated alterations in intestinal barrier function may contribute to variability in corticosteroid disposition and therapeutic response (Jönsson et al., 1995; Zhang et al., 2018).

#### 3.2.1 Interaction between corticosteroids and gut microbiota

Beyond host-mediated processes, studies have revealed interplay between CSs and the gut microbiota. CSs may undergo microbial biotransformation, including activation and degradation by specific gut microorganisms (Yadav et al., 2013), while corticoid therapy itself can induce shifts in microbial composition in animal models and human cohorts (Li et al., 2023b; Lyu et al., 2025). However, compared with other UC therapies, microbiota-related evidence for corticosteroids remains relatively limited (Becker et al., 2023). In pediatric UC, corticosteroid treatment has been associated with dynamic changes in the gut microbiota. Higher baseline microbial diversity and enrichment of *Oscillospiraceae* (*Ruminococcaceae*) were associated with better treatment response, whereas prolonged CSs exposure was linked to dysbiosis characterized by increased abundance of *Pseudomonadota* and reduced SCFA-producing taxa (Lazarević et al., 2025). Similarly, Blesl et al. (2024) reported that baseline microbiota had limited predictive value, while responders exhibited longitudinal restoration of beneficial taxa such as *Blautia, Anaerostipes*, and *Bifidobacterium*, in contrast to non-responders who showed minimal changes and an increase in *Streptococcus salivarius*. Additional evidence summarized by Becker et al. (2023) further highlighted that corticosteroid-related microbiota data remain heterogeneous. *Ex vivo* exposure to methylprednisolone reduced the amount of *Bifidobacterium longum*, whereas budesonide treatment in patients with microscopic colitis increased *Oscillospiraceae* and *Ruminococcus_2*, but decreased *Faecalibacterium* abundance, alongside higher within-patient diversity. Collectively, these findings suggest that corticosteroid therapy is accompanied by dynamic microbiota remodeling, whereas it remains unclear whether these alterations contribute directly to therapeutic efficacy or primarily reflect resolution of intestinal inflammation.

Although CSs remain an important component of UC management, concerns regarding steroid dependency, prolonged exposure, and treatment-related adverse effects, together with the increasing availability of fast-acting biologics and small-molecule therapies, have promoted a shift toward steroid-sparing treatment strategies (Selinger et al., 2017; Feuerstein et al., 2025).

#### 3.2.2 Interplay between probiotics and corticosteroids

The gut microbiota has been shown to participate in corticosteroid biotransformation. However, it remains unknown whether probiotic supplementation can influence corticosteroid absorption, metabolism, or systemic exposure, given that pharmacokinetic interactions between probiotics and corticosteroids have not been systematically investigated. Although recent experimental evidence suggests that selected probiotic formulations may modulate the activity of certain cytochrome P450 enzymes (Zhang et al., 2024a), there is currently no direct evidence that these effects alter the pharmacokinetics of corticosteroids. Likewise, no evidence suggests that probiotics substantially affect 11β-HSD1 or the major drug transporters involved in corticosteroid disposition (Lee et al., 2024). Consequently, the beneficial effects of probiotic supplementation are currently thought to arise predominantly through complementary pharmacodynamic mechanisms rather than altered corticosteroid pharmacokinetics. Nevertheless, several *Lactobacillus* and *Bifidobacterium* strains, as well as multi-strain probiotic preparation the De Simone Formulation have been shown to improve intestinal barrier function and attenuate mucosal inflammation by enhancing the expression of tight-junction proteins, including occludins, claudins, and zonula occludens-1 (ZO-1), thereby reducing intestinal permeability and restoring epithelial integrity (Cheng et al., 2020; Di Vito et al., 2022; You et al., 2025). In parallel, probiotics can influence immune signaling through increased production of anti-inflammatory mediators such as IL-10 and suppression of pro-inflammatory cytokines including TNF-α, IL-6, IL-12, and IL-1β (Mazziotta et al., 2023; Guo and Lv, 2023). Consistent with these immunomodulatory mechanisms, administration of the De Simone formulation in patients with active UC promoted a more tolerogenic colonic dendritic-cell phenotype together with enhanced IL-10-associated responses, further supporting the ability of selected probiotic formulations to modulate key immune pathways relevant to corticosteroid therapy (Ng et al., 2010).

Direct evidence evaluating concomitant probiotic–corticosteroid administration remains extremely limited. In a randomized study performed in healthy dogs, co-administration of the multi-strain probiotic Visbiome with prednisone did not reduce glucocorticoid-induced gastric bleeding or mucosal injury compared with prednisone alone, indicating that this probiotic formulation did not provide additional gastroprotective effects in this experimental setting (Rak et al., 2023). Nevertheless, preclinical studies suggest that probiotics may mitigate selected systemic adverse effects associated with glucocorticoid therapy. Inceu et al. (2023) demonstrated that co-administration of a *Bacillus* spore-based probiotic partially attenuated dexamethasone-induced metabolic, inflammatory, oxidative, and histological disturbances in rats. In a subsequent study, co-administration of *Saccharomyces boulardii* or *Lacticaseibacillus paracasei* with dexamethasone attenuated glucocorticoid-induced metabolic disturbances, including hyperglycemia, systemic inflammation, and hepatic steatosis (Inceu et al., 2025). Although these studies were conducted outside the IBD setting, they suggest that selected probiotics may help mitigate metabolic and inflammatory adverse effects of glucocorticoid therapy. However, whether these benefits extend to patients with UC or influence corticosteroid efficacy or pharmacokinetics remains unknown. Beyond their potential effects on glucocorticoid-associated adverse effects, current evidence is insufficient to determine whether probiotic supplementation directly modifies corticosteroid efficacy or pharmacokinetics in UC. Existing data primarily support the concept that selected probiotic strains exert immunomodulatory and barrier-protective effects through mechanisms overlapping with corticosteroid action, thereby providing a biological rationale for future mechanistic and clinical studies evaluating adjunctive probiotic use during corticosteroid therapy.

### 3.3 Immunomodulators

Immunomodulators occupy an intermediate position in the UC therapeutic algorithm and are mainly used to reduce corticosteroid exposure, maintain remission, or provide rapid rescue immunosuppression in selected severe cases (Danese et al., 2020). In UC, this group primarily includes thiopurines such as azathioprine and 6-MP, which are used mainly as steroid-sparing maintenance agents, and calcineurin inhibitors (CIs) such as cyclosporine A and tacrolimus, which are reserved for acute severe or steroid-refractory disease (Sebastian et al., 2024). As these drugs act through systemic immunomodulation and are associated with clinically significant adverse effects and interindividual variability in efficacy and toxicity, their relationship with the gut microbiota is attracting increasing interest, particularly with regard to microbial metabolism, response to treatment, and potential interactions with probiotic or postbiotic interventions.

#### 3.3.1 Interaction between immunomodulators and gut microbiota

As the principal representatives of thiopurines, 6-MP and its prodrug azathioprine are key immunomodulating agents used in UC management (Zakerska-Banaszak et al., 2022). The mechanism of action of thiopurines involves the incorporation of their pharmacologically active metabolite, 6-thioguanine nucleotides (6-TGN), into DNA or RNA as false purine analogs, which lead to DNA damage, cell cycle arrest and apoptosis, and inhibition of nucleotide and protein synthesis (González-Lama and Gisbert, 2016). Because of their delayed therapeutic effect, thiopurines are often initiated during active UC alongside corticosteroids and subsequently used as steroid-sparing maintenance therapy in steroid-dependent or steroid-refractory patients (Berends et al., 2019). Interindividual variability in thiopurine efficacy and toxicity is largely driven by genetic polymorphisms in TPMT and NUDT15, necessitating individualized dosing and regular hematological and hepatic monitoring (Moriyama et al., 2016; Singh and Sood, 2026). Although thiopurine metabolism has traditionally been attributed to host enzymes, accumulating evidence indicates that the gut microbiota also participates in azathioprine biotransformation (Lazarević et al., 2022). Experimental evidence supports the role of the gut microbiota in thiopurine disposition. Using a pseudo-germ-free rat model generated by broad-spectrum antibiotic treatment, Wang et al. (2022) demonstrated that depletion of the gut microbiota significantly reduced systemic exposure to the active metabolite 6-TGN, with a 34% decrease in AUC_0–t_ and a 35% reduction in C_max_ compared with specific pathogen-free controls. Exposure to 6-methylmercaptopurine also showed a decreasing trend. Notably, these pharmacokinetic changes were not accompanied by alterations in TPMT or NUDT15 genotype, hepatic TPMT or xanthine oxidase expression, or 6-TGN clearance, suggesting a contribution of the gut microbiota to thiopurine disposition. *In vitro* experiments further confirmed that intestinal microbial enzymes can directly convert azathioprine into 6-MP, 6-TGN, and 6-methylmercaptopurine, demonstrating that gut bacteria participate in both the activation and inactivation of thiopurines. Furthermore, *Blautia wexlerae* was shown to express a selenium-dependent xanthine dehydrogenase capable of converting 6-MP into inactive 6-thioxanthine, thereby reducing azathioprine efficacy in murine colitis models, whereas administration of *Bacillus subtilis* partially restored treatment responsiveness by suppressing microbial 6-MP inactivation (Yan et al., 2023). Conversely, other bacterial species have been reported to generate active thioguanine nucleotides from thiopurine precursors, while *Pseudomonadota* may encode TPMT-like methyltransferases that redirect thiopurine metabolism toward inactive metabolites (Franzin et al., 2021; Bartocci et al., 2026). In addition to these direct metabolic interactions, thiopurines themselves may alter gut microbial composition and exert antimicrobial activity against selected IBD-associated microorganisms, including *Campylobacter concise* and *Mycobacterium avium* subsp. *paratuberculosis*, while also affecting mucosal bacterial adherence (Liu et al., 2017). Conversely, studies in patients with Crohn’s disease indicate that thiopurines do not markedly alter gut microbial composition but instead influence its metabolic activity and functional profile (Becker et al., 2026; Favaron et al., 2026). Taken together, these findings highlight a potential role of microbiota-mediated metabolism in thiopurine disposition and may represent an additional source of interindividual variability in thiopurine efficacy beyond established host pharmacogenetic factors (Caenepeel et al., 2020; Lazarević et al., 2022). Nevertheless, the clinical relevance of this interaction in patients with UC remains to be fully elucidated.

The second group of immunomodulators includes CIs, primarily cyclosporine A and tacrolimus, which are used as rescue therapy in patients with acute severe or steroid-refractory UC. These agents suppress T-cell activation through inhibition of calcineurin-mediated IL-2 signaling and exhibit pharmacokinetic variability related to CYP3A metabolism and P-glycoprotein-mediated transport (Nogimori et al., 2024; Kunasol et al., 2025). While CIs are effective for inducing remission in patients with steroid-refractory UC, their use is limited by the need for therapeutic drug monitoring and the risk of significant adverse effects, including nephrotoxicity and infections (Berends et al., 2019; Dancewicz et al., 2025). Increasing evidence suggests that the immunomodulatory effects of CIs may also contribute to alterations in gut microbiota composition (Kunasol et al., 2025). In UC mouse models, tacrolimus improved gut dysbiosis, as reflected by an increased relative abundance of genera such as *Lactobacillus*, *Allobaculum*, and *Adlercreutzia*, alongside a reduction in taxa including *Bilophila*, *Mucispirillum*, and *Escherichia* (Lv et al., 2023). Current evidence indicates that interactions between CIs and the gut microbiota are bidirectional but remain insufficiently characterized. Experimental studies suggest that gut bacteria may influence tacrolimus metabolism, while CIs themselves can modulate microbial composition and function, including SCFA-associated pathways (Becker et al., 2023; O’Reilly et al., 2023). Taken together, these findings suggest that microbiota-drug interactions may contribute to interindividual variability in the therapeutic response to CIs, although their clinical relevance in patients with UC remains to be clarified.

#### 3.3.2 Interplay between probiotics and immunomodulators

Pharmacokinetic interactions between probiotics and thiopurines, particularly azathioprine and 6-MP, remain largely unexplored. Experimental evidence suggests that probiotic bacteria and microbiota-derived metabolites may influence thiopurine disposition through effects on intestinal absorption and microbial biotransformation. Using a Parallel Artificial Membrane Permeability Assay (PAMPA), Danić et al. (2025) demonstrated that selected *Lactobacillus* and *Bifidobacterium* strains, together with the microbial metabolite deoxycholate, altered azathioprine permeability across artificial intestinal membranes. Increased apparent permeability was accompanied by reduced recoverable drug concentrations, suggesting bacterial uptake, sequestration, or biotransformation of azathioprine. Although these findings were obtained under *in vitro* conditions, they support the concept that microbial activity within the intestinal lumen may affect the fraction of thiopurine available for systemic absorption. However, despite the growing recognition of microbiota-mediated thiopurine metabolism, no clinical studies have systematically evaluated the impact of probiotic supplementation on azathioprine or 6-MP pharmacokinetics in patients with UC. This lack of clinical evidence may partly reflect the complexity of thiopurine metabolism, which is influenced by multiple host and microbial factors, making probiotic-specific effects difficult to isolate in clinical settings.

Similar to thiopurines, evidence regarding probiotic-CIs interactions is currently limited and derived predominantly from preclinical studies. In a DSS-induced colitis model, co-treatment with *Lactiplantibacillus plantarum* 550 enhanced the therapeutic efficacy of tacrolimus, resulting in improved clinical and histopathological outcomes, reduced intestinal inflammation, and favorable remodeling of the gut microbiota and modulation of bile acid metabolism, reflected by downregulation of bile acid-sensing receptors TGR5 and FXR, and increased cecal taurochenodeoxycholic acid concentrations. These beneficial effects were further accompanied by downregulation of multiple immune- and inflammation-associated pathways, including IFN-*γ* and IFN-α responses and IL2–STAT5 signaling (Lv et al., 2023). Evidence from bone marrow transplantation demonstrated that co-administration of *Lactobacillus acidophilus* with tacrolimus upregulated T-reg genes such as *Nt5e, Foxp3, Ikzf2, Nrp1* and *Itgb8* in CD4 + T cells, suppressed Th17 differentiation, and alleviated graft-versus-host disease while maintaining the efficacy and safety of immunosuppressive therapy (Baek et al., 2022). Although these findings originate from a non-IBD setting, they further support the concept that selected probiotic strains may complement CIs therapy through modulation of host immune responses in a strain- and disease-specific manner. In contrast, another experimental study found that the addition of probiotics to cyclosporin A therapy did not consistently improve anti-inflammatory outcomes beyond those achieved by cyclosporin A treatment alone (Dosh et al., 2024). Moreover, a recent study by Tsuchiya et al. (2025) suggests that probiotic–CIs interactions may also involve pharmacokinetic mechanisms. Among the 11 probiotic strains tested, *Bacillus subtilis TO-A* exhibited the highest capacity to degrade tacrolimus and everolimus *in vitro*, reducing their residual concentrations by approximately 8% and 17%, respectively. Consistent with these findings, oral administration of *B. subtilis* TO-A in mice significantly decreased systemic tacrolimus exposure, resulting in significant reduction in peak blood concentration (C_max_) compared with control animals. These observations indicate that orally administered microorganisms may metabolize or inactivate CIs before their absorption in the small intestine, potentially reducing drug bioavailability and therapeutic efficacy. Importantly, the narrow therapeutic index of tacrolimus makes such reductions in systemic exposure clinically relevant, as they may result in subtherapeutic drug concentrations (Lloberas et al., 2025). These findings therefore warrant caution regarding concomitant supplementation with *Bacillus*-containing probiotics, as altered tacrolimus exposure could potentially compromise therapeutic efficacy. Although confirmation in humans is still lacking, these findings highlight the need for caution when co-administering tacrolimus with probiotic preparations and emphasize the importance of evaluating strain-specific pharmacokinetic interactions in future clinical studies (Tsuchiya et al., 2025). Taken together, currently available evidence indicates that probiotic–CI interactions are highly strain-, drug-, and context-dependent and may involve both pharmacodynamic and pharmacokinetic mechanisms. While selected probiotic strains may enhance the therapeutic effects of CIs through complementary immunoregulatory mechanisms, others may reduce drug exposure through microbial metabolism. Nevertheless, current evidence remains limited to isolated preclinical and non-IBD studies, and the clinical relevance of these interactions in patients with UC has yet to be established. The scarcity of clinical investigations may partly reflect concerns regarding administration of live probiotics to patients receiving potent immunosuppressive therapy, despite their generally favorable safety profile (Abraham and Quigley, 2020).

### 3.4 Biological therapies

Biologic therapies represent a major advance in the management of moderate-to-severe UC and are increasingly used in patients who fail to respond adequately to conventional treatment or require therapeutic escalation. Currently available biologics target key inflammatory pathways involved in UC pathogenesis and include anti-TNF agents (infliximab, adalimumab, and golimumab), anti-integrin therapy (vedolizumab), and inhibitors of IL-12/23 or IL-23 signaling (ustekinumab, mirikizumab, and guselkumab) (Danese et al., 2014; Thadiboina et al., 2025). Owing to their ability to induce and maintain remission while reducing corticosteroid exposure, biologics have become a cornerstone of contemporary UC treatment. Given that biologics are administered parenterally and are degraded through proteolytic mechanisms, direct microbiota-mediated effects on drug absorption and metabolism are considered less relevant than for orally administered therapies. Nevertheless, accumulating evidence suggests that the gut microbiota may contribute to variability in therapeutic response through modulation of host immune and inflammatory pathways (Rodríguez-Moranta et al., 2024).

#### 3.4.1 Interactions between biologic therapies and gut microbiota

The strongest evidence, among biologic therapies, is currently available for anti-TNF agents and vedolizumab. Several clinical observational studies have demonstrated that patients achieving clinical remission exhibit distinct baseline microbial profiles compared with non-responders supporting the concept that the gut microbiota may influence therapeutic efficacy (Malinauskiene et al., 2025; Hu et al., 2024; Tamburini et al., 2024; Sakurai et al., 2020; Wang et al., 2018; Magnusson et al., 2016). Most consistently, responders generally display lower baseline dysbiosis, higher microbial diversity, and enrichment of beneficial commensals, particularly *F. prausnitzii*. Other taxa repeatedly linked to favorable outcomes include *Akkermansia muciniphila*, *Ruminococcus* spp., and members of the families *Lachnospiraceae* and *Oscillospiraceae*, whereas *Eggerthellaceae Pseudomonadota*, *Verrucomicrobiota* and other inflammation-associated microorganisms are more frequently observed in non-responders (Liu et al., 2023; Tamburini et al., 2024; Wang et al., 2018). Both baseline and post-treatment analyses identified *F. prausnitzii* and the newly recognized *Flavonifractor plautii* as taxa associated with clinical remission (Tamburini et al., 2024).

Furthermore, longitudinal studies indicate that microbiota dynamics are closely associated with the long-term efficacy of biologic therapy in UC. In patients receiving adalimumab, clinical remission was accompanied by progressive remodeling of the gut microbiota, characterized by enrichment of beneficial genera, including *Bifidobacterium*, *Dorea*, together with distinct baseline microbial signatures differentiating remitters from non-remitters (Oh et al., 2024). Similarly, longitudinal metagenomic study of vedolizumab-treated patients demonstrated that sustained remission was associated with greater microbiota stability and enrichment of anti-inflammatory, SCFA-producing commensals, including *Bacteroides uniformis*, *Roseburia* spp. (*R. intestinalis*, *R. inulinivorans*, *R. faecis*), and members of the *Lachnospiraceae* family. In contrast, non-responders exhibited persistent dysbiosis together with greater taxonomic and functional heterogeneity, suggesting that microbiota stability may be a prerequisite for effective anti-integrin therapy (Alonso et al., 2025). Recent multi-omics approaches further support the predictive value of the gut microbiota. In a prospective multicenter study, integration of fecal and mucosal microbial signatures significantly improved prediction of infliximab response compared with clinical variables alone, highlighting the potential of the gut microbiota as a predictive biomarker and a potential modulator of treatment response in UC (Chen et al., 2026b).

Although these findings are promising, several limitations remain. Most available studies involve relatively small cohorts, use heterogenous sequencing and analytical approaches, and are frequently confounded by dietary and lifestyle factors. In addition, many studies combine patients with Crohn’s disease and UC, limiting the identification of UC-specific microbial signatures. Notably, microbiota-based models have demonstrated greater predictive accuracy for treatment response than conventional clinical variables alone (Meade et al., 2023). Nevertheless, microbiota-focused research has remained largely restricted to anti-TNF agents and vedolizumab. Clinical studies of newer biologics have primarily evaluated therapeutic efficacy, whereas microbiota-related investigations remain scarce. Evidence regarding ustekinumab is derived predominantly from studies in Crohn’s disease rather than UC (Doherty et al., 2018), while corresponding data for mirikizumab and guselkumab are currently lacking. Given the increasing use of these newer biologics in UC management, defining microbiota-related predictors of response and elucidating potential microbiota-mediated mechanisms of action should be considered a key priority for future research. Current evidence collectively indicates that the gut microbiota represents both a promising biomarker and a potential biological modulator of response to biologic therapy in UC. However, larger prospective, longitudinal and mechanistic studies are needed before microbiome-informed therapeutic decision-making can be implemented in routine clinical practice.

#### 3.4.2 Interplay between biologics and probiotics

In the context of biologic therapies administered concomitantly with probiotic supplementation, direct pharmacokinetic interactions are less straightforward, as monoclonal antibodies do not undergo classical intestinal metabolism. Nevertheless, modulation of inflammatory activity, intestinal barrier integrity, and microbial homeostasis may influence drug loss through the damaged mucosa, immunogenicity, and the overall persistence of therapeutic response (Yang et al., 2026). The co-administration of probiotics in patients with UC receiving biologic therapy represents a promising adjunctive strategy that may favorably affect gut microbial composition, intestinal barrier function, and the regulation of mucosal immune responses (Iheozor-Ejiofor et al., 2020). Clinical studies evaluating concurrent probiotic supplementation during biologic therapy in UC are currently lacking, despite this compelling biological rationale. Available evidence is derived almost exclusively from experimental models, which consistently suggest that selected probiotic strains may enhance the therapeutic efficacy of biologic therapy through complementary immunomodulatory and microbiota-mediated mechanisms. In DSS-induced colitis models, co-administration of *Bifidobacterium longum* CECT 7894 with infliximab significantly improved clinical and histopathological outcomes compared with infliximab monotherapy. The combination restored intestinal barrier integrity through increased expression of tight-junction proteins, enriched beneficial taxa including *Bifidobacterium* and *Blautia*, and increased the production of secondary bile acids with anti-inflammatory properties (Xiao et al., 2022). Similarly, *Lactobacillus gasseri* potentiated the therapeutic effects of infliximab by reducing the expression of pro-inflammatory cytokines (TNF-α, IFN-*γ*, IL-6, and IL-17A), increasing IL-10 production, improving epithelial barrier function, and promoting expansion of *Prevotella* spp. (Han et al., 2022). Comparable findings have been reported using the next-generation probiotic *Bacteroides thetaiotaomicron*, whose combination with infliximab resulted in greater attenuation of colitis severity than either treatment alone. This effect was accompanied by modulation of genes involved in epithelial integrity and mucus production, including upregulation of *Ocln*, *Tff3*, and *Muc2*, together with downregulation of *Ace2* (Badi et al., 2025). These preclinical studies collectively indicate that probiotic supplementation may enhance the efficacy of anti-TNF therapy through complementary mechanisms involving suppression of intestinal inflammation, restoration of epithelial barrier function, remodeling of the gut microbiota, and increased production of beneficial microbial metabolites, including SCFAs and secondary bile acids. However, these observations have not yet been validated in clinical studies, and no pharmacokinetic investigations have evaluated whether probiotics influence biologic drug exposure or immunogenicity in patients with UC.

Although direct clinical evidence for probiotic supplementation during biologic therapy in UC is unavailable, microbiota-targeted combination approaches are beginning to emerge. An open-label pilot study combining fecal microbiota transplantation with vedolizumab demonstrated the feasibility and safety of simultaneously targeting the gut microbiota and immune cell trafficking, supporting the concept that modulation of the gut microbiome may complement biologic therapy (Syed et al., 2026). However, the exploratory nature of the study, together with its small sample size and recruitment limitations, underscores the need for larger controlled trials to establish clinical efficacy. Together, these findings support the concept that targeted modulation of the gut microbiota may represent a complementary strategy for optimizing biologic therapy, which requires further evaluation in well-designed randomized clinical trials.

### 3.5 Small molecule therapies

The development of orally administered small-molecule therapies has expanded treatment options for moderate-to-severe UC, particularly in patients with inadequate response to conventional therapies or biologics (Herrera-deGuise et al., 2023). Currently approved agents include Janus kinase (JAK) inhibitors (tofacitinib, upadacitinib, and filgotinib) and sphingosine-1-phosphate (S1P) receptor modulators (ozanimod and etrasimod) (Sakurai and Saruta, 2026). Although both effectively suppress intestinal inflammation, they act through distinct mechanisms: JAK inhibitors inhibit intracellular cytokine signaling via the JAK/STAT pathway, whereas S1P receptor modulators reduce lymphocyte trafficking by preventing lymphocyte egress from lymph nodes (Nemeth et al., 2017; Lin et al., 2020; Zou et al., 2023).

#### 3.5.1 Interaction between small molecules and gut microbiota

Emerging evidence suggests that small-molecule therapies may interact with the gut microbiota through both direct and indirect mechanisms. In an *in vitro* human colonic model, Favaron et al. (2024) demonstrated that several IBD drugs, including JAK inhibitors and S1P receptor modulators, exhibited variable stability in the presence of fecal microbiota, with reductions in drug concentration observed following microbial exposure. These findings suggest that the gut microbiota may influence the luminal fate of small-molecule drugs before systemic absorption and raise the possibility that interindividual differences in microbial composition contribute to variability in therapeutic response (Al-Btoosh et al., 2026). In contrast, clinically relevant concentrations of tofacitinib did not significantly alter microbial diversity, community structure, SCFAs production, or metabolic activity in the artificial intestinal model M-SHIME®, indicating that its therapeutic effects are mediated predominantly through host immune signaling rather than direct microbiota modulation (Favaron et al., 2026). To date, no animal or clinical studies have demonstrated that S1P receptor modulators directly alter gut microbial composition. However, experimental evidence suggests that intestinal S1P signaling itself may influence the gut microbiota. Miao et al. (2022) demonstrated that SphK2 deficiency increased intestinal S1P signaling, resulting in enrichment of *Bacillota (Firmicutes)* and *Verrucomicrobiota*, altered conjugated bile acid profiles, and downstream metabolic disturbances. Thus, pharmacological modulation of the S1P pathway may exert indirect microbiota-mediated effects beyond immune cell trafficking.

#### 3.5.2 Interplay between probiotics and small molecules

Currently, evidence regarding probiotic interactions with JAK inhibitors and S1P receptor modulators remains virtually absent. To date, neither preclinical nor clinical studies have systematically evaluated the effects of probiotic supplementation on the pharmacokinetics, efficacy, or safety of these orally administered small-molecule therapies. Nevertheless, experimental evidence indicates that microbiota-targeted interventions, including selected probiotic strains and fecal microbiota transplantation, can modulate inflammatory pathways converging on JAK/STAT signaling, whereas microbiota-derived metabolites may contribute to the anti-inflammatory effects of JAK inhibition (Panganiban et al., 2025; Zang et al., 2024). These findings provide a biological rationale for adjunctive probiotic supplementation alongside small-molecule therapies in UC. Clinical relevance of this approach, however, remains hypothetical and requires validation in well-designed preclinical and clinical studies. Interestingly, a recent study extended microbiota-derived therapeutic strategies to small-molecule therapy by developing an engineered outer membrane vesicle (OMV)-based delivery system for upadacitinib. Dai et al. (2026) encapsulated upadacitinib within *Escherichia coli* Nissle-derived OMVs and demonstrated enhanced intestinal retention and colon-targeted drug release. In mouse models of acute and chronic UC, this approach combined JAK–STAT inhibition with OMV-mediated restoration of intestinal barrier integrity, modulation of the gut microbiota, and macrophage polarization, resulting in enhanced anti-inflammatory and anti-fibrotic effects. Although this study does not represent a conventional interaction between a live probiotic and upadacitinib, it provides proof-of-concept that microbiota-derived vesicles can be integrated with small-molecule therapies to achieve complementary therapeutic effects.

## 4 Toward rational probiotic use in UC

Recent advances in microbiome research suggest that probiotic therapy in UC should evolve from empirical supplementation toward more rational and precision microbiota-based strategies tailored to individual microbiome characteristics, functional microbial deficits, and specific therapeutic objectives rather than diagnosis alone. Several general principles emerge from the available literature that may guide future integration of probiotics into UC management. As summarized in Figure 2, rational probiotic therapy should be guided by several complementary principles, including evidence-based strain and formulation selection, clearly defined therapeutic objectives, optimization of treatment timing and duration, and consideration of the pharmacological therapy administered. The following sections discuss each of these principles in greater detail.

**Figure 2:**
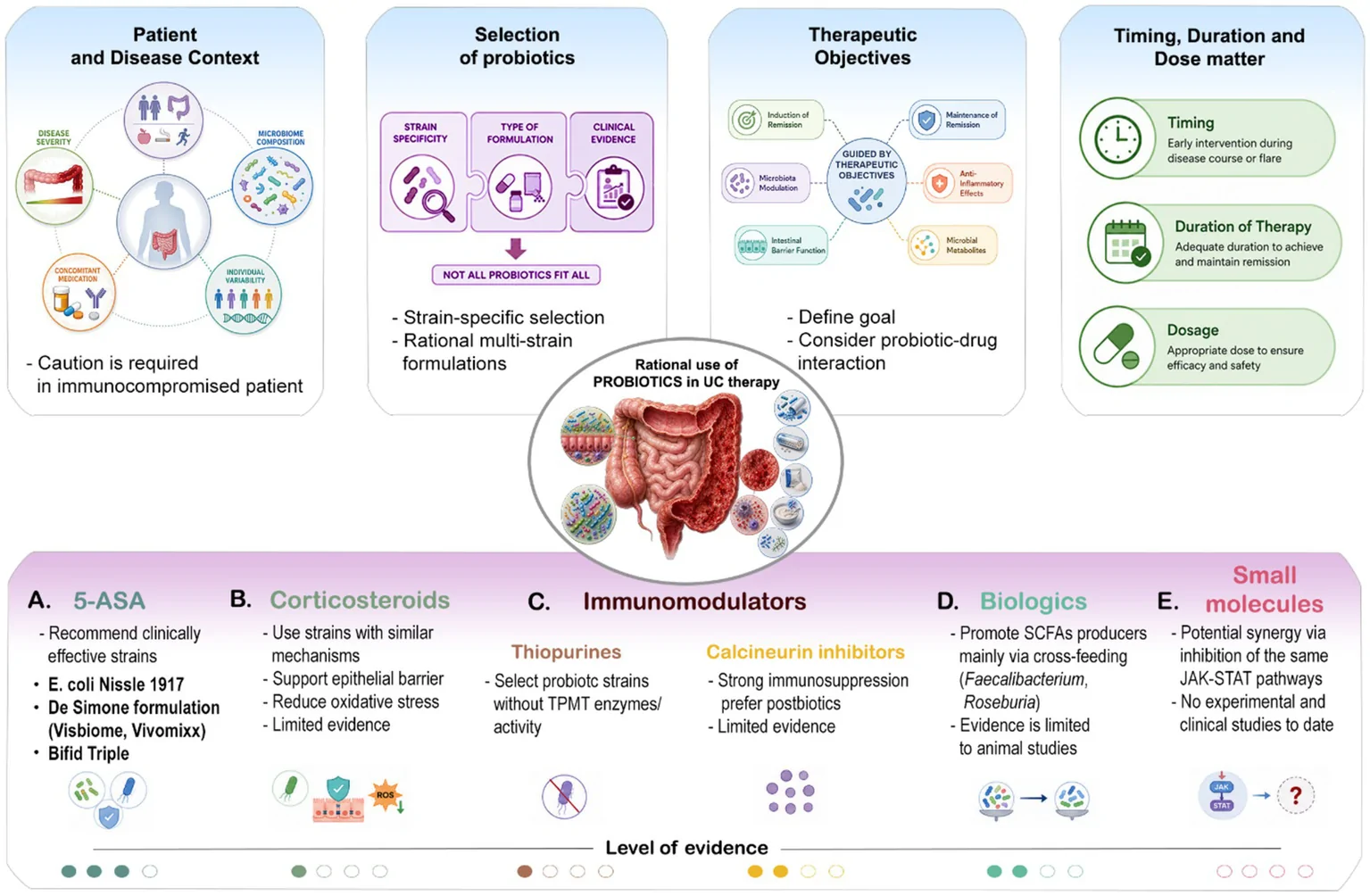
Proposed framework for rational probiotic use in ulcerative colitis. The proposed framework summarizes key considerations for evidence-based integration of probiotics and postbiotics into UC management. Selection of microbiota-directed interventions should be guided by patient and disease characteristics, evidence-based probiotic formulation, therapeutic objectives, and optimization of treatment timing, duration, and dose. Current evidence indicates that probiotic recommendations should be individualized according to the concomitant pharmacotherapy, reflecting the distinct pharmacokinetic and pharmacodynamic microbiota-drug interactions. The strongest evidence supports adjunctive probiotic use with selected 5-aminosalicylate regimens, whereas evidence for corticosteroids, immunomodulators, biologics, and small-molecule therapies remains limited, variable, and drug-specific. This framework illustrates a transition from empirical probiotic supplementation toward precision, mechanism-based microbiota-directed therapy. The relative level of evidence is indicated by filled circles (●), based on a qualitative assessment of the available evidence summarized in this review. 5-ASA, 5-aminosalicylic acid; JAK, Janus kinase; ROS, reactive oxygen species; SCFAs, short-chain fatty acids; STAT, signal transducer and activator of transcription; TPMT, thiopurine S-methyltransferase; UC, ulcerative colitis.

First, not all probiotics should be considered therapeutically equivalent and probiotic efficacy should not be extrapolated, even within the same bacterial species. Clinical efficacy is highly strain- and formulation-specific, and only a limited number of well-characterized preparations, have demonstrated consistent benefits in patients with UC (Kruis et al., 2004; Bibiloni et al., 2005; Sood et al., 2009; McFarland et al., 2018). Thus, probiotic selection should be evidence-based rather than assuming a class effect across commercially available preparations. Moreover, rationally designed probiotic combinations may outperform single strains by promoting metabolic cross-feeding between lactate- and butyrate-producing bacteria (Qiao et al., 2025; Chen et al., 2026c).

Second, probiotic supplementation should be guided by the therapeutic objective rather than by the diagnosis alone. Different treatment settings may require distinct microbiota-directed strategies, including restoration of microbial homeostasis during active inflammation, maintenance of remission, prevention of therapy-associated dysbiosis, enhancement of mucosal barrier function, or support of microbial metabolic activity. Future probiotic interventions in UC should, therefore, focus less on the enrichment of individual taxa and more on restoration of beneficial microbial functions, including SCFAs production, maintaining epithelial barrier integrity, regulating the bile acid metabolism and supporting immunoregulatory pathways.

Probiotic supplementation should likewise be tailored to both the administered therapy and the timing and duration of treatment, as these factors may influence therapeutic outcomes (Liu et al., 2025; Niu et al., 2026). During active disease, probiotics may primarily facilitate restoration of epithelial barrier integrity and attenuation of inflammation, whereas prolonged administration during remission may be more appropriate for maintaining microbial homeostasis and reducing the risk of relapse.

The choice of probiotic intervention should also be guided by the mechanism of action of the administered drug. For patients receiving 5-ASA, selected clinically validated probiotic preparations, such as *Escherichia coli* Nissle 1917, the De Simone formulation, or Bifid Triple Viable Capsules, may be considered as adjunctive therapy, as current evidence supports complementary pharmacodynamic mechanisms involving restoration of microbial homeostasis, reinforcement of epithelial barrier integrity, and modulation of mucosal immune responses. During corticosteroid therapy, selected probiotic formulations may be considered as adjunctive interventions to support epithelial barrier restoration, reduce oxidative stress, and modulate mucosal immune responses. However, current evidence is insufficient to support routine use, and further well-designed clinical studies are required to establish their efficacy and optimal timing during corticosteroid induction therapy. When immunomodulatory therapies are used, rational probiotic selection should consider both the metabolic properties of individual probiotic strains and the degree of immunosuppression. During thiopurine therapy, adjunctive probiotics should ideally restore intestinal homeostasis without interfering with microbial pathways involved in thiopurine metabolism, although current evidence remains insufficient to support strain-specific recommendations. By contrast, patients receiving potent immunosuppressive therapies, particularly CIs, routine administration of live probiotics should be approached cautiously because of limited clinical evidence and potential safety concerns in profoundly immunocompromised individuals. In these settings, postbiotics may provide safer alternatives while preserving many of the beneficial immunomodulatory and barrier-protective effects attributed to live microorganisms. Moreover, the recent demonstration of strain-specific microbial metabolism of tacrolimus further supports careful selection of adjunctive probiotic formulations in patients receiving CIs (Tsuchiya et al., 2025; Cooper and Bhushan, 2024). For patients treated with biologic agents, particularly anti-TNF therapies, growing evidence indicates that successful therapeutic response is associated with restoration of microbial diversity and enrichment of beneficial commensals, including *Faecalibacterium* and *Roseburia* (Schierová et al., 2021). These observations provide a strong rationale for developing next-generation probiotics based on functionally relevant commensal microorganisms, characteristic of treatment-responsive patients rather than conventional probiotic preparations with limited disease-specific activity. Nevertheless, most of these candidates are strict anaerobes, posing substantial manufacturing and formulation challenges, and their long-term safety and clinical efficacy remain to be established (Jan et al., 2024).

Ultimately, integration of microbiome profiling with pharmacomicrobiomics, metabolomics, and clinical phenotyping may enable rational selection of the most appropriate probiotic intervention for each patient (Doestzada et al., 2018; Jiang et al., 2016; Goldiș et al., 2025). Together with advances in next generation probiotics, postbiotics, BEVs, and functionally designed probiotic consortia capable of restoring key microbial metabolic functions, such as butyrate production through metabolic cross-feeding, these approaches may represent the next generation of microbiota-based adjunctive therapies for UC (Inokuma et al., 2025).

## 5 Knowledge gaps and future perspectives

Despite the therapeutic promise of microbiota-guided interventions, several important challenges must be addressed before these strategies can be translated into routine clinical practice. Both conventional therapies and microbiota-directed interventions can influence treatment outcomes in UC through complex microbiota-host interactions. However, the majority of available studies have focused primarily on clinical endpoints, disease activity, and taxonomic changes in microbial composition, whereas the underlying mechanisms responsible for these effects remain insufficiently characterized (Tian et al., 2020; Xiao et al., 2022).

One of the most significant knowledge gaps concerns the limited understanding of pharmacokinetic interactions between probiotics and UC therapies. While experimental studies conducted outside the IBD research demonstrate that probiotics may alter drug absorption, metabolism, bioavailability, and efficacy, comparable evidence for UC medications remains scarce (Purdel et al., 2023). Most studies evaluating probiotics in UC have focused on clinical efficacy without investigating whether observed benefits are mediated through changes in drug disposition, microbial biotransformation, host signaling pathways, or their combination. Importantly, these evidence gaps are particularly pronounced for more recently introduced UC therapies. Although preclinical studies provide some evidence of probiotic interactions with selected biologic agents, clinical data remain scarce, particularly for therapies such as ustekinumab, mirikizumab, and guselkumab. The gap is even greater for small-molecule therapies, for which direct evidence on interactions with live probiotics remains virtually absent despite their increasing use in UC. Although a recent preclinical study demonstrated the feasibility of combining probiotic-derived BEVs with upadacitinib in a targeted delivery platform (Dai et al., 2026), whether live probiotics modify the pharmacokinetics or pharmacodynamics of these agents remains unknown, highlighting an important priority for future mechanistic and clinical research.

In addition, many studies continue to emphasize alterations in microbial composition, although it is increasingly recognized that microbial function related to metabolic changes may be more relevant than taxonomic abundance alone. Distinct microbial communities can perform similar metabolic functions, whereas identical taxa may exert different biological effects depending on strain-specific properties and host context (Jiang et al., 2016). Future studies should increasingly focus on functional microbiome characteristics, including microbial metabolite production, immune modulation, epithelial barrier regulation, and host–microbe signaling networks. This transition from taxonomic profiling toward functional microbiome characterization represents one of the key prerequisites for future precision microbiota-guided therapy. The integration of multiomic-approaches, particularly metabolomics, metagenomics, transcriptomics, and pharmacomicrobiomics may provide important insights into the mechanisms linking microbiota alterations with therapeutic response (Chetty and Blekhman, 2024). Such approaches could help identify functional biomarkers associated with treatment efficacy, remission maintenance, and response to microbiota-targeted interventions.

Another emerging area is the development of postbiotic therapies, including BEVs, which may overcome several limitations associated with live probiotic administration. Given that probiotics consist of live microorganisms, their administration during intensive immunosuppressive therapy warrants careful consideration. This may partly explain the scarcity of clinical studies evaluating probiotic-immunomodulator interactions and highlights the potential value of alternative approaches, including BEVs, which may retain immunomodulatory activity without requiring the administration of viable bacteria (Abraham and Quigley, 2020). In this context, postbiotics may offer potential advantages related to standardization, safety, stability, and regulatory control (Qian et al., 2025). As non-living and non-replicating biological entities, BEVs can be purified, quantified, and administered at defined particle concentrations, potentially facilitating product standardization and quality control based on vesicle size, cargo composition, and biological activity. Their characterization is increasingly guided by Minimal Information for Studies of Extracellular Vesicles (MISEV)-based analytical frameworks, supplemented by BEV-specific quality-control considerations (Zhang et al., 2024b; Razim et al., 2024). Unlike live probiotics, BEVs do not require maintenance of cellular viability and can exert biological activity independently of bacterial colonization, potentially offering advantages in terms of storage, handling, and product consistency. Moreover, their non-replicating nature avoids risks directly associated with the administration of viable microorganisms, including bacterial translocation, systemic infection, and uncontrolled colonization. This may be particularly relevant for immunocompromised patients, in whom impaired intestinal barrier function and immunosuppressive therapy may increase susceptibility to probiotic-related adverse events (Olovo et al., 2024; Chen et al., 2026d; Mairal et al., 2026). Furthermore, the possibility of engineering BEVs to modify their cargo or reduce undesirable microbial components may provide additional opportunities to optimize their safety and therapeutic properties (Chen et al., 2026a).

Nevertheless, evidence supporting their use in UC remains limited to animal experiments, and further research is needed to identify the postbiotic preparations, metabolites, or microbial components most relevant for disease management and therapeutic modulation (Hao et al., 2021; Nie et al., 2025). Moreover, their potential to modulate the efficacy or pharmacokinetics of UC therapies remains largely unexplored.

The development of probiotic-assisted drug delivery systems and functionally designed microbial consortia capable of enhancing prodrug activation or restoring key microbial metabolic functions represents another promising direction for precision microbiota-guided pharmacotherapy (Sahoo et al., 2025; Zhao et al., 2025). Future efforts should therefore focus on identifying reproducible functional biomarkers that can guide rational selection of probiotics in conjunction with specific UC therapies. Integration of microbiome profiling with pharmacomicrobiomics, metabolomics, artificial intelligence, and clinical phenotyping may ultimately enable selection of the most appropriate microbiota-targeted intervention for individual patients. Artificial intelligence and machine-learning approaches capable of integrating microbiome, metabolomic, pharmacological, and clinical datasets are expected to play an increasingly important role in identifying predictive biomarkers and guiding individualized microbiota-based therapies (Kumar et al., 2026).

Personalized microbiota-guided therapeutic strategies represent an attractive concept, although their implementation in routine clinical practice remains limited by several challenges (Goldiș et al., 2025). These include an incomplete understanding of remission-associated microbial and metabolic signatures, substantial interindividual variability in microbiome composition and function, lack of standardized analytical approaches, and the cost and limited accessibility of advanced multi-omics technologies. Additional challenges arise from the translation of precision probiotic interventions into standardized clinical products. Rigorous manufacturing and quality-control procedures are required to ensure strain identity, purity, viability, stability, and batch-to-batch consistency (Slijkerman et al., 2025). Moreover, large-scale production, formulation, production costs, and heterogeneous regulatory requirements may complicate commercialization, particularly for next-generation probiotics (Kim et al., 2026). Nevertheless, UC represents a particularly attractive indication for precision microbiota-guided therapy because of its chronic relapsing–remitting course. As maintaining long-term remission remains one of the primary therapeutic goals, individualized microbiota-directed interventions may provide sustained benefits over prolonged periods and thereby contribute to improved long-term disease control.

Addressing these challenges will be essential for translating advances in pharmacomicrobiomics and microbiome research into mechanism-based, microbiota-informed therapeutic strategies capable of improving treatment efficacy and long-term disease control in UC. Future studies should focus on elucidating strain-specific probiotic-drug interactions, identifying reproducible functional biomarkers of therapeutic response, and validating microbiota-guided therapeutic strategies in well-designed prospective clinical trials. Such trials should integrate metagenomic and metabolomic analyses and consider stratification according to baseline microbiome characteristics to identify functional biomarkers of response and determine whether probiotic efficacy differs among specific patient subgroups.

## 6 Conclusion

Selected probiotic formulations are already incorporated into the management of UC, but their future clinical application should increasingly rely on precision microbiota-based strategies rather than empirical supplementation. Rational selection of probiotics should integrate the patient’s microbial and metabolic profile, disease stage, therapeutic objective, concomitant pharmacotherapy, and probiotic-specific characteristics, including strain, formulation, dose, and treatment duration. Together with next-generation probiotics, postbiotics such as BEVs, and other microbiota-derived therapeutics, these approaches may further optimize the efficacy of conventional therapies and complement the expanding therapeutic landscape of biologics and small-molecule drugs in UC. However, achieving this goal will require well-designed mechanistic and clinical studies to elucidate probiotic-drug interactions, particularly for newer biologic agents and small-molecule therapies, where current evidence remains remarkably limited.
